# Excellent ballistic impact resistance of Al_0.3_CoCrFeNi multi-principal element alloy with unique bimodal microstructure

**DOI:** 10.1038/s41598-021-02209-y

**Published:** 2021-11-22

**Authors:** Saideep Muskeri, Bharat Gwalani, Shristy Jha, Anqi Yu, Philip A. Jannotti, Ravi Sankar Haridas, Brian E. Schuster, Jeffrey T. Lloyd, Rajiv S. Mishra, Sundeep Mukherjee

**Affiliations:** 1 Department of Materials Science and Engineering, University of North Texas, Denton, TX 76203 USA; 2 Advanced Materials and Manufacturing Processes Institute, University of North Texas, Denton, TX 76207 USA; 3 Physical and Computational Sciences Directorate, Pacific Northwest National Laboratory, Richland, WA 99352 USA; 4 DEVCOM Army Research Laboratory, Aberdeen Proving Ground, Aberdeen, MD 21005 USA; 5 Department of Metallurgical, Materials and Biomedical Engineering, University of Texas at El Paso, El Paso, TX 79968 USA

**Keywords:** Engineering, Materials science

## Abstract

Multi-principal element alloys represent a new paradigm in structural alloy design with superior mechanical properties and promising ballistic performance. Here, the mechanical response of Al_0.3_CoCrFeNi alloy, with unique bimodal microstructure, was evaluated at quasistatic, dynamic, and ballistic strain rates. The microstructure after quasistatic deformation was dominated by highly deformed grains. High density of deformation bands was observed at dynamic strain rates but there was no indication of adiabatic shear bands, cracks, or twinning. The ballistic response was evaluated by impacting a 12 mm thick plate with 6.35 mm WC projectiles at velocities ranging from 1066 to 1465 m/s. The deformed microstructure after ballistic impact was dominated by adiabatic shear bands, shear band induced cracks, microbands, and dynamic recrystallization. The superior ballistic response of this alloy compared with similar Al_x_CoCrFeNi alloys was attributed to its bimodal microstructure, nano-scale L1_2_ precipitation, and grain boundary B2 precipitates. Deformation mechanisms at quasistatic and dynamic strain rates were primarily characterized by extensive dislocation slip and low density of stacking faults. Deformation mechanisms at ballistic strain rates were characterized by grain rotation, disordering of the L1_2_ phase, and high density of stacking faults.

## Introduction

Multi-principal element alloys (MPEAs) or high entropy alloys (HEAs) represent a new paradigm in structural alloy development based on five or more elements in equimolar or near equimolar proportions^1–4^. These alloys tend to form single or multiple solid solution phases^5^ and possess promising physical and mechanical properties such as high strength and ductility, good fracture toughness, high hardness, excellent work hardenability, superior strain rate sensitivity, good fatigue, and wear resistance^6^. Low strain rate (in the range of 10^−4^ s^−1^–10^−2^ s^−1^) and dynamic strain rate (in the range of 10^1^ s^−1^–10^3^ s^−1^) response of HEAs has been extensively studied for the fundamental understanding of their plastic deformation mechanisms^7–10^. Previous studies focused on single phase HEAs have demonstrated that the deformation mechanisms, strain hardening, flow stress, and strain rate sensitivity are strongly influenced by the strain rate^11–13^, owing to the unique combination of solid solution strengthening, twinning, dislocation mediated plasticity, and stress-induced phase transitions^14^. Al_x_CoCrFeNi alloys have been widely studied as a model system due to their desirable microstructure, ranging from single-phase to multi-phase solid solutions, obtained with a slight change in aluminum content^5,15–18^. Al_0.3_CoCrFeNi (x = 0.3) is one such system with single-phase face centered cubic (FCC) crystal structure in its as-cast condition but showed precipitation of intermetallic phases of B2 and L1_2_ after thermomechanical processing. This alloy exhibited excellent mechanical properties through Hall–Petch and precipitate strengthening mechanisms^15,18^. A wide variety of microstructures has been reported for this alloy as a function of thermomechanical treatments including, single phase FCC, FCC + B2, and FCC + L1_2_^14,15,18,19^. This shows that Al_0.3_CoCrFeNi HEA has an enormous scope for attaining microstructural complexity through different thermomechanical processing routes, which in turn makes it an excellent model alloy system for tuning its mechanical properties. There have been several studies focused on the deformation mechanisms in Al_x_CoCrFeNi alloys, but there is a lack of systematic analysis of the deformation mechanism as a function of strain rate in compositionally and microstructurally complex alloys.

The deformation mechanism in several HEAs has been reported to transition from dislocation-mediated to twinning-induced plasticity with increase in strain rate. Twin boundaries act as barriers for dislocation movement and increase the strain hardenability^11^. The ability for twinning depends on the stacking fault energy (SFE), which for several FCC-based HEAs has been reported to be in the range of 20 to 30 mJ/m^2^^20^. In low-SFE HEAs, dislocations dissociate into partials with large separation^21^ and require more thermal activation for cross-slip resulting in an increase in strain rate sensitivity (SRS)^22^. However, there is limited understanding of the microstructure evolution at high strain rates in the range of 10^3^ s^−1^–10^5^ s^−1^. Application of HEAs in high-speed loading conditions requires in-depth knowledge of their dynamic deformation behavior^23^, such as in ballistic impact and penetration^24^.

Here, the response of thermomechanically processed Al_0.3_CoCrFeNi HEA was studied as a function of wide range of strain rates. It had a unique bimodal microstructure composed of FCC matrix with nano-sized L1_2_ precipitates and grain boundary B2 precipitates. This alloy was chosen because of its unique and complex microstructure engineered towards a good combination of high strength and enhanced ductility. The deformation behavior and failure modes were studied in quasistatic tensile and bulk compression, Split-Hopkinson pressure bar (SHPB) tests, and ballistic impact. The microstructural response and failure modes were examined for detailed insights into deformation mechanisms at the different strain rates.

## Experimental

### Alloy making

The Al_0.3_CoCrFeNi (in atomic ratio) alloy was obtained from ACI Alloys (San Jose, California, USA) in the form of a plate, 12 mm in thickness, made from constituent elements with > 99.9% purity. The plate was homogenized by hot rolling (HR) to 70% at 620^0^C and subsequent annealing at the same temperature for 100 h under argon atmosphere followed by water quenching. The surface was mechanical grinded to obtain uniform roughness and flatness.

### Mechanical characterization

Quasi-static (strain rate of 10^–3^ s^−1^) mini-tensile experiments were performed using a computer-controlled mini-tensile testing frame containing a 500-pound load cell with specimen dimensions of gauge length ~ 5 mm and width ~ 1 mm. Samples were sequentially polished on both sides using SiC papers and subsequently on cloth to a final thickness of ~ 0.8 mm to achieve mirror finish. Compression tests at strain rates of 10^–3^, 10^–2^, and 10^–1^ s^−1^ were conducted using a MTS 322 load frame equipped with a 500 kN load cell. An initial pre-load of 1 kN was applied to eliminate any compliance of the compression jaws. High strain rate compression experiments were performed using a Split Hopkinson Pressure Bar (SHPB) from REL Inc SureTest Systems at strain rates of 700, 1200, and 1750 s^−1^. All the compression tests were performed at room temperature on cubic specimens having a side length of 4 mm. At least three samples were tested at each strain rate for statistical analysis. The specimen surfaces in contact with compression jaws were lubricated with Vaseline to avoid any errors due to inhomogeneity. The compression experiments at low strain rates (10^–3^, 10^–2^, and 10^–1^ s^−1^) and high strain rate (700, 1200, and 1750 s^−1^) were recorded at a sampling rate of 10 Hz and 1 MHz, respectively. High speed nanoindentation measurements were done using a Triboindenter (Bruker, Minneapolis, USA) for hardness mapping. A diamond Berkovich indenter at a peak load of 10 mN with loading and unloading rates of 2 mN/s and a hold time of 2 s was used for indentation mapping. The neighboring indents were separated by ~ 200 μm to avoid overlap of their plastic zones and to capture the hardness maps with high resolution over a large area^11,25^.

### Ballistic test

Ballistic testing was conducted at the DEVCOM Army Research Laboratory (Aberdeen Proving Ground, Maryland, USA) using 6.35 mm WC spherical projectiles fired from a 7.62 mm diameter smooth-bore powder gun at varying velocities ranging from 1066 to 1465 m/s. Three shot conditions with different velocities, namely, 1066 m/s (low), 1340 m/s (intermediate), and 1465 m/s (high) were used to investigate the failure modes under ballistic impact. The selected shot conditions were sectioned across the crater using an electrical discharge machine (EDM) for microstructural analysis.

### Microstructural characterization

The samples were initially polished using SiC abrasive papers followed by alumina and colloidal silica to a surface roughness of 0.02 μm for mirror finish. Pre- and post-deformation microstructural characterization was performed using scanning electron microscopy (SEM) and electron backscattered diffraction (EBSD) technique using FEI Nova Nano SEM230 equipped with a Hikari Super EBSD detector. The EBSD data was further interpreted using orientation imaging microscopy (OIM) software. X-ray diffraction (XRD) analysis was performed using Rigaku Ultima X-ray diffractometer by Cu-Kα radiation with a wavelength of 1.54 Å. An aberration-corrected transmission electron microscope (Titan 80-300 S/TEM from Thermo Fisher Scientific, USA) equipped with a high-angle annular dark-field (HAADF) detector and an energy dispersive spectroscopy (EDS) system was employed at 300 kV for bright-field (BF), dark-field (DF), high resolution transmission electron microscopy (HRTEM), HAADF scanning TEM (STEM), selected area electron diffraction (SAED), and composition analysis.

## Results and discussion

### Alloy microstructure

**Figure 1:**
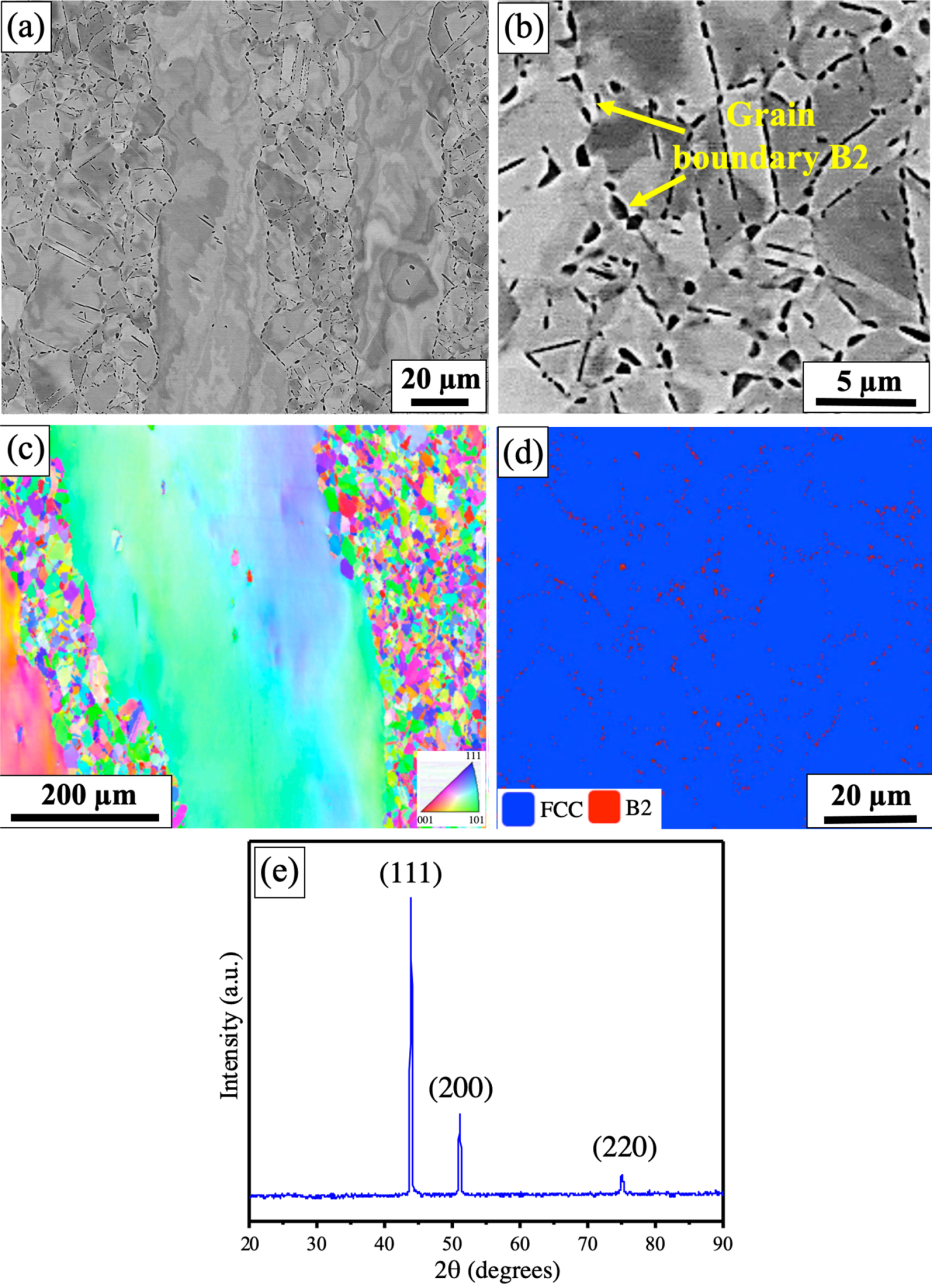
Microstructural characterization of thermomechanically processed Al_0.3_CoCrFeNi high entropy alloy: (**a**) backscattered electron (BSE) microscopy image showing bimodal microstructure of elongated strained grains and recrystallized fine grains; (**b**) A high magnification BSE image showing B2 precipitates decorating the grain boundaries; (**c**) EBSD inverse pole figure showing bimodal grains with recrystallized fine grains of average size ~ 15 µm; (**d**) A high magnification EBSD phase map showing FCC and B2 phases in blue and red colors, respectively; (**e**) X-ray diffraction analysis showing FCC crystal structure.

The microstructure of the thermomechanically processed Al_0.3_CoCrFeNi HEA alloy is shown in Fig. 1a, consisting of bimodal microstructure with some recrystallized fine grains from high temperature annealing and strained elongated coarse grains from hot rolling. Grain boundary precipitates (black contrast) were also seen at high magnification backscattered electron (BSE) microscopy image shown in Fig. 1b, previously identified to be a B2 phase^15^. These precipitates were observed predominantly in the recrystallized fine-grained regions. Figure 1c shows the low magnification inverse pole figure (IPF) indicating bimodal grain morphology. The recrystallized grains showed an average grain size of ~ 15 μm. A high-magnification EBSD phase map, shown in Fig. 1d, confirmed the presence of majority FCC phase (blue color) and grain boundary B2 precipitates (red color) with volume fraction of ~ 98% and 2%, respectively. X-ray diffraction analysis showed peaks corresponding to FCC crystal structure (Fig. 1e). Peaks corresponding to the B2 phase were not observed probably due to their small volume fraction in the overall microstructure.

**Figure 2:**
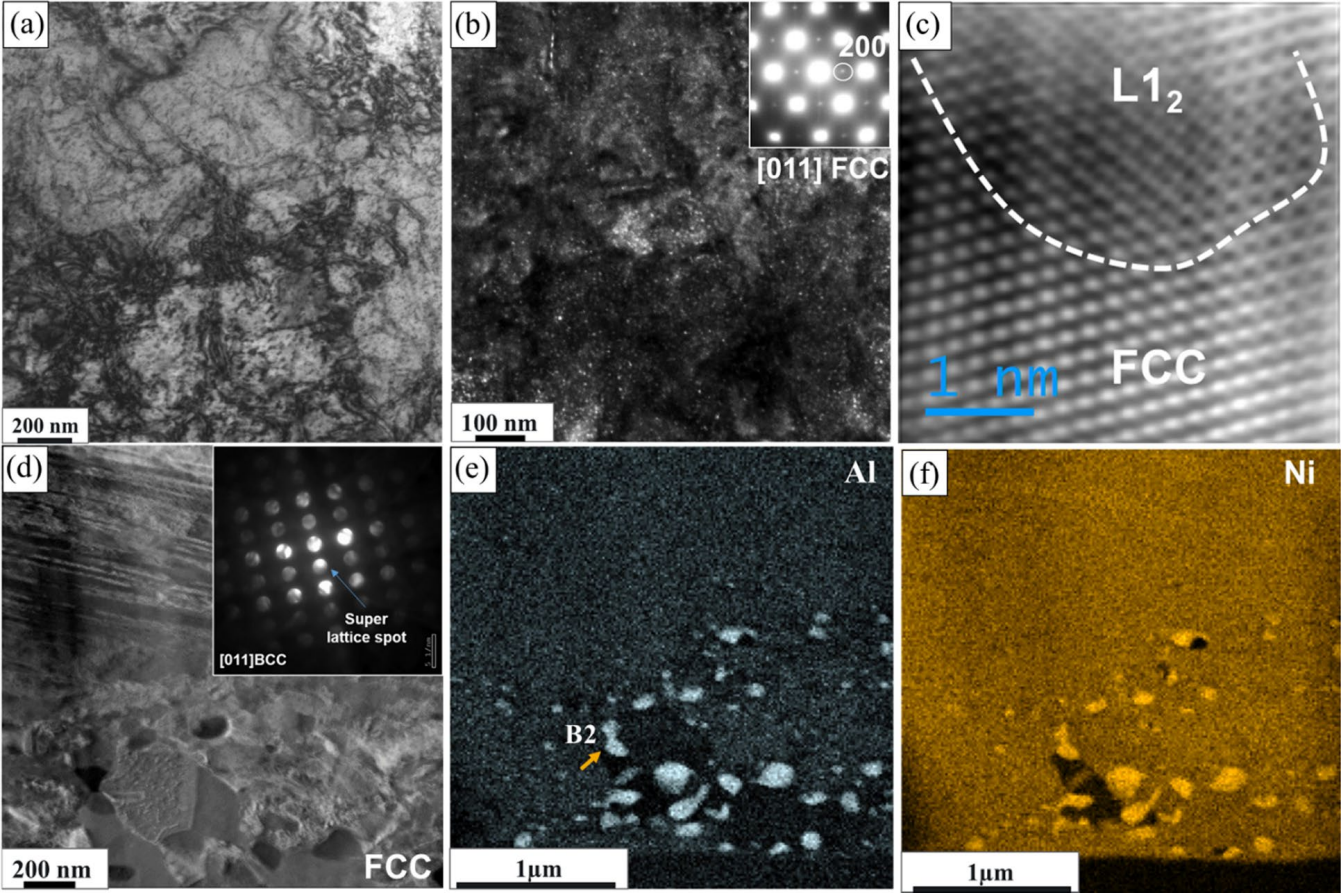
TEM characterization of the thermomechanically processed Al_0.3_CoCrFeNi high entropy alloy: (**a**) Bright field TEM image showing high density of dislocations from the hot rolling; (**b**) high magnification dark field TEM image with the inset showing TEM SADP recorded from [011]_fcc_ zone axis indicating L1_2_ ordering in the matrix; (**c**) HRTEM image showing continuity across the ordered and disordered FCC phases; (**d**) BF-TEM image taken from near the grain boundary showing precipitates and the SADP (inset) confirming the structure of the precipitates to be ordered B2; (**e**) and (**f**) TEM EDS from the grain boundary region confirming (Al, Ni)-rich B2 precipitates.

TEM characterization of the thermomechanically processed Al_0.3_CoCrFeNi HEA alloy is shown in Fig. 2. Figure 2a represents the BF-TEM image showing an FCC grain with many dislocations from the hot rolling. The DF-TEM image created from {200} superlattice spot in Fig. 2b shows distribution of ordered L1_2_ precipitates (~ 5 nm) in the FCC matrix. The faint superlattice reflections at {01–1} and {200} position in the [011]_fcc_ diffraction pattern (inset of Fig. 2b), where a {200} spot is encircled in the figure, unambiguously shows ordering in the FCC matrix. At a higher resolution (HRTEM) in Fig. 2c, the phase contrast image depicting the atomic columns further demonstrates continuity across the ordered and disordered FCC phases. This suggests minimal lattice misfit between the two phases^26^. The image in Fig. 2d was taken from near the grain boundary region which was decorated with relatively larger (~ 100–500 nm) spherical precipitates and recrystallized FCC grains. The inset in the figure shows the selected area diffraction pattern (SADP) from [011]_bcc_ zone axis (ZA) from one such spherical precipitate exhibiting super lattice reflections and revealing ordered BCC (B2) precipitates. Corresponding STEM-EDS maps of Al and Ni from the region, shown in Fig. 2e and f respectively, further confirmed (Al, Ni)-rich B2 precipitates in the FCC matrix. The thermomechanical processing resulted in a unique combination of bimodal FCC grains with homogeneously distributed nano-scale L1_2_ phase and heterogeneously formed B2 precipitates along the grain boundaries of the recrystallized finer grains.

### Mechanical behavior at quasi-static and dynamic strain rates

**Figure 3:**
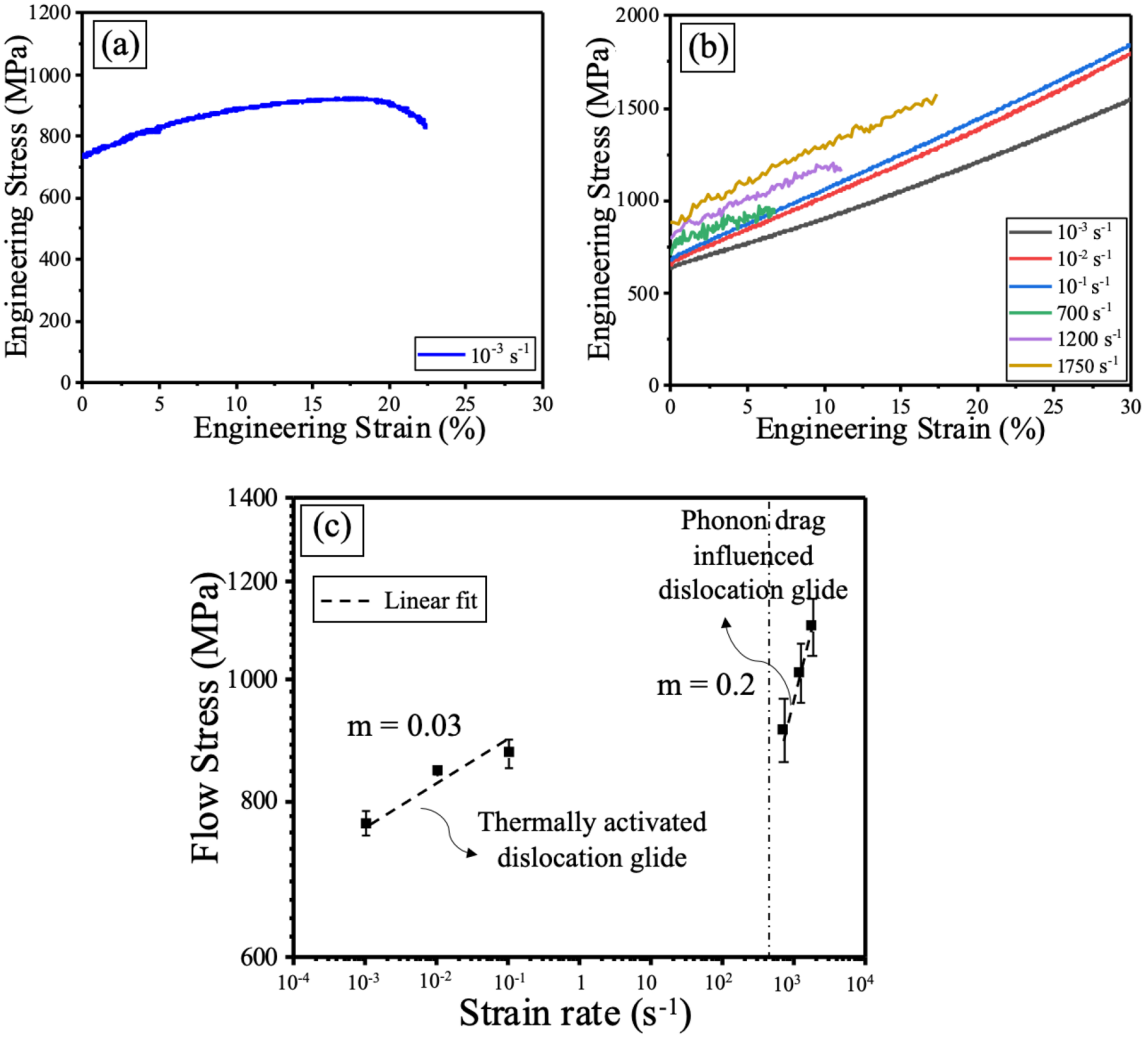
Mechanical behavior at quasistatic and dynamic strain rates: (**a**) quasistatic tensile engineering stress–strain curve; (**b**) quasistatic and dynamic strain rate compressive engineering stress–strain curves; (**c**) flow stress versus strain rate to determine strain rate sensitivity of the alloy at two different strain rate regimes.

Quasistatic tensile engineering stress–strain curve for the alloy is shown in Fig. 3a. The alloy exhibited good combination of strength and ductility with a tensile yield strength (σ_0.2_) of 734.2 ± 15.8 MPa, ultimate tensile strength (σ_UTS_) of 921.3 ± 18.9 MPa, and elongation of ~ 21.2 ± 3.5%. The compressive engineering stress–strain curves, for both low and high strain rates, are shown in Fig. 3b. The average compressive yield strength (σ_0.2_) at quasistatic and dynamic strain rates are summarized in Table 1. The end of flow stress in the compression curves for the dynamic tests indicates the end of loading pulse on the split-Hopkinson bar, rather than failure of the samples. The continuous absorption of impact energy is evident from the monotonic increase in the compressive yield strength of the alloy with increase in strain rate. The alloy showed high strain hardenability at both low and high strain rates as seen from Fig. 3b. For example, the quasi-static compressive yield strength was ~ 700 MPa; but the flow stress reached up to 1500 MPa at about 35% plastic strain. A similar dependence of strength on strain rate was previously reported for other FCC HEAs with low stacking fault energy (SFE) such as Al_0.1_CoCrFeNi^8^ and Al_0.7_CoCrFeNi^27^. The excellent mechanical properties of the present alloy may be attributed to several factors. First, the addition of Al to CoCrFeNi results in high lattice strain due to the large atomic size difference between Al and the other elements in this alloy^8,15^. This lattice strain reduces the SFE, which directly influences its strain hardenability^28^. Hence, during deformation, full dislocations are easily dissociated into partials and these partials in turn prevent cross-slip, even at low strain rates. The large fraction of partial dislocations significantly increases the strain hardening ability^8,15^. Second, the grain boundary precipitates in this alloy inhibit dislocation motion and create back stress leading to further increase in strength^29^. Third, the fine L1_2_ precipitates in the matrix lead to strengthening by particle shearing mechanism^30^. Finally, the bimodal microstructure leads to a good combination of strength and ductility^31,32^. The fine grains contribute towards strengthening by inhibiting dislocation motion while the coarse grains deform extensively resulting in overall increase in percentage elongation. Strain rate sensitivity (SRS), *m*, is a measure of the dependence of strength on strain rate and was evaluated as^13^:

**Table 1:** Average compressive yield strength at quasistatic and dynamic strain rates.

Strain rate	Quasistatic			Dynamic		
	10^–3^ s^−1^	10^–2^ s^−1^	10^–1^ s^−1^	700 s^−1^	1200 s^−1^	1750 s^−1^
Yield strength (MPa)	703.8 ± 18.5	722.7 ± 6.3	725.3 ± 16.4	794.2 ± 54.4	828.2 ± 55.7	902.7 ± 57.9

1 $$m=\frac{\partial \mathrm{ln}\sigma }{\partial \mathrm{ln}\dot{\epsilon }}$$

where, *σ* is the flow stress and $\dot{\varepsilon }$ is strain rate. Compressive yield strength was calculated at 5% strain and plotted on a double-logarithmic scale of flow stress (MPa) versus strain rate (s^−1^) as shown in Fig. 3c. SRS of two different regimes was obtained from the slope of a linear fit as shown by the dotted lines. The SRS was 0.03 for low strain rates (10^–3^ s^−1^–10^–1^ s^−1^) while it was about an order of magnitude higher with a value of 0.2 in the high strain rate (10^2^ s^−1^–10^3^ s^−1^) regime. At low strain rates, dislocation velocities are small and that leads to negligible effect of phonon drag on dislocation motion^8^. Therefore, flow stress changes at a slower rate with change in strain rate and mostly depends on thermally activated dislocation glide^8^. The SRS value of our alloy is larger compared to other single-phase FCC HEAs reported in literature, such as Al_0.1_CoCrFeNi^8^ and CoCrFeMnNi^23^. The high SRS value at high strain rates may be attributed to two factors: (i) not enough time for thermally activated dislocations to cross the energy barrier and (ii) strong effect of viscous phonon drag on dislocation motion^27^. The frequency of vibration of lattice atoms (phonons) is not high enough to interfere with dislocation motion at low strain rates. In contrast, the phonon drag effect on dislocation motion is more pronounced at high strain rates and leads to significant increase in yield strength with increase in strain rate^23^.

### Microstructural characterization after deformation at quasistatic and dynamic strain rates

**Figure 4:**
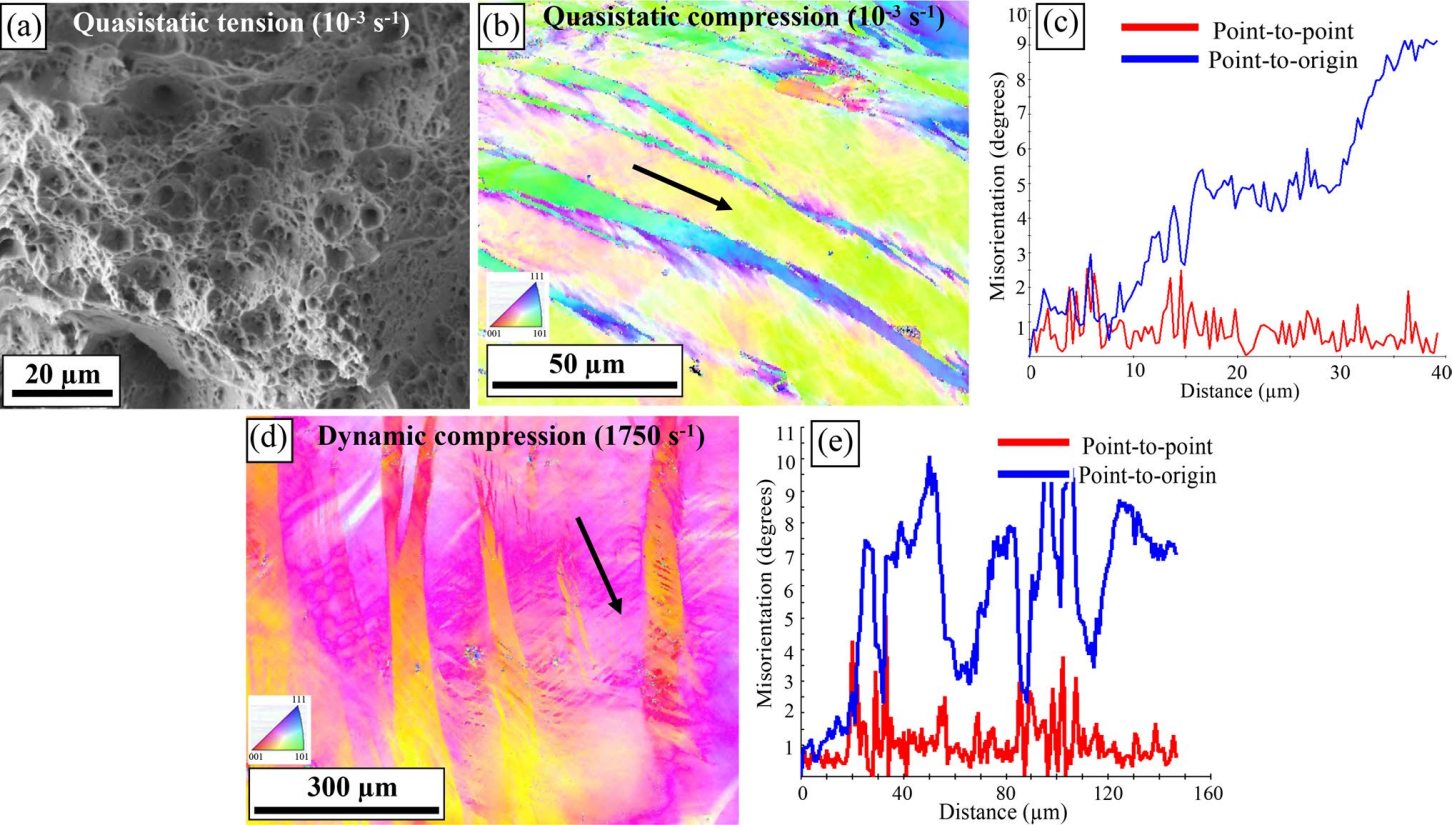
Microstructural characterization after deformation at quasi-static and dynamic strain rates: (**a**) fracture surface after quasi-static tensile test showing ductile mode of fracture; (**b**) IPF image of the deformed microstructure after quasi-static compression test showing deformed grains with deformation bands; (**c**) misorientation profile along the arrow in Fig. 3b indicating an average misorientation of ~ 5–6 degrees; (**d**) IPF image of the deformed microstructure after SHPB test at a strain rate of 1750 s^−1^ showing highly deformed grains and high density of deformation bands; (**e**) misorientation profile along the arrow in Fig. 3d indicating an average misorientation of ~ 7–8 degrees.

The fracture morphology after quasistatic tensile test, shown in Fig. 4a, revealed uniformly distributed voids and shallow dimples of different sizes. Many of these voids were coalesced together indicating a ductile type of failure mode. IPF image in Fig. 4b shows the deformed microstructure after quasistatic strain-rate compression (at 10^–3^ s^−1^) indicating highly deformed grains with deformation/slip bands. The misorientation profile, shown in Fig. 4c along the arrow in Fig. 4b, indicates that the average misorientation of the deformation bands was ~ 5–6 degrees. Figure 4d shows the IPF map of the deformed microstructure of the alloy after SHPB test at the strain rate of 1750 s^−1^. The microstructure showed severely deformed grains with high density of deformation bands. The color gradient seen within the deformed grains for both the quasi-static and dynamic tests represent orientation gradient attributed to slip^33^. Figure 4e shows the misorientation profile along the arrow shown in Fig. 4d. The deformation bands had an average misorientation of ~ 7–8 degrees. The alloy showed high strain hardening at the strain rate of 1750 s^−1^ (Fig. 3b) without any signs of adiabatic shear bands (ASBs) or crack formation in the deformed microstructure. This indicates that the material resisted any localized failure and exhibited good shear resistance. Shear localization takes place when thermal softening due to temperature rise generated during plastic work is insufficient to overcome the rate of strain hardening. Previously, the resistance to shear localization for single phase Al_0.3_CoCrFeNi alloy, at high strain rates was attributed to the excellent strain hardenability from forest dislocation hardening, mechanical twinning, and solid solution strengthening^14^. Also, in low-SFE HEAs, the distribution of SFE is inhomogeneous since chemical ordering of constituent elements varies throughout the lattice^34^. Due to this complex energy landscape, the deformation mechanism may vary and lead to simultaneous twinning and microband formation during deformation^34^. Note that in our current examination, we did not notice any signature of micro- or nano-scale twinning that has been previously reported. In the present case, the microstructure consists of grain boundary B2 precipitates, nano-scale L1_2_ precipitates in the matrix, and bimodal distribution of grains. Therefore, the SFE landscape may be fairly complex and not promote twin formation^35^.

**Figure 5:**
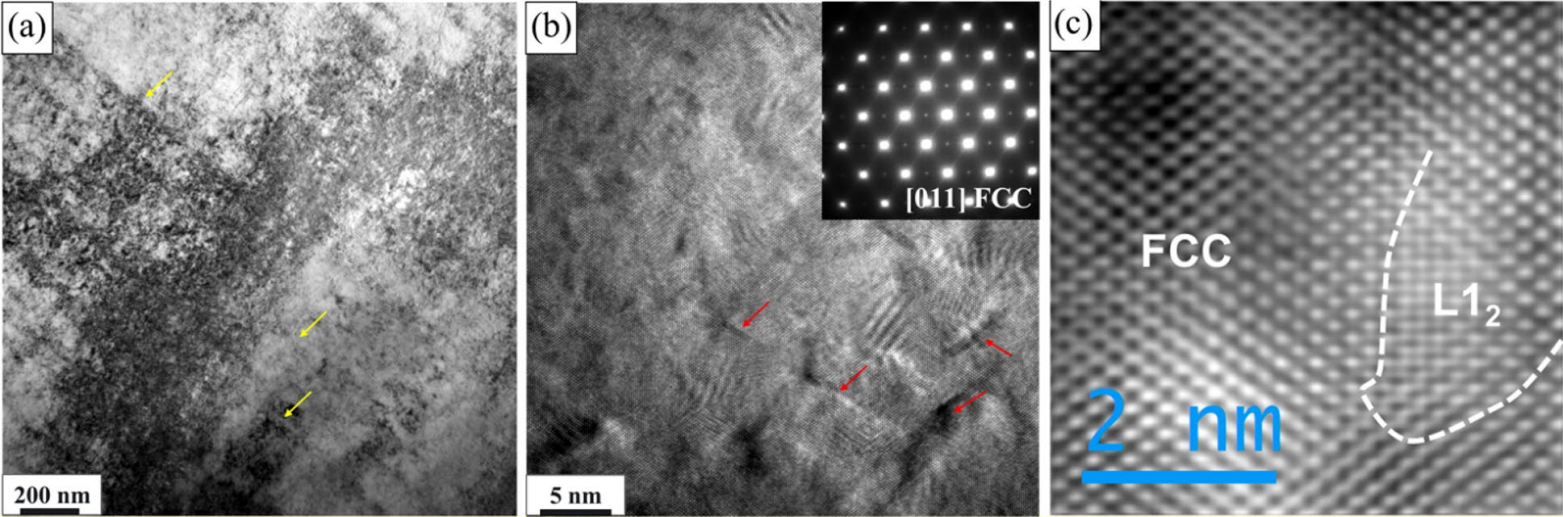
TEM characterization of the sample after deformation at dynamic strain rate of 1750 s^−1^: (**a**) BF-TEM image showing aligned bands of high density of dislocations (marked by yellow arrows); (**b**) HRTEM image showing extensive slip and limited stacking fault density (marked by red arrows) with the inset showing SADP pattern taken from [011]_FCC_ ZA which represents the L1_2_ superlattice reflections; (**c**) HRTEM image showing continuity across the FCC/L1_2_ interface after the high strain rate deformation.

To further understand the dynamic deformation behavior during SHPB testing, TEM characterization was done for the sample deformed with the highest strain rate (1750 s^−1^). Figure 5a shows high density of dislocations and deformation bands aligned in one direction (marked by yellow arrows) indicating dislocation slip to be the main deformation mechanism at this strain rate. HRTEM image in Fig. 5b shows low density of stacking faults (marked by red arrows). The inset in Fig. 5b shows superlattice spots in the SADP from [011]_FCC_ ZA, indicating the presence of L1_2_. Figure 5c further shows the FCC/L1_2_ boundary, confirming that the nano-scale precipitates were intact during the dynamic deformation of the sample. Hence, even without any sign of micro- or nano-scale twinning, the excellent work hardening shown by this alloy during dynamic deformation may be attributed to solid solution strengthening, grain boundary hardening due to the presence of fine recrystallized FCC grains in the microstructure, and precipitation strengthening from the grain boundary B2 precipitates and nano-scale L1_2_ precipitates^30,35,36^.

### Ballistic impact and hardness contour maps

**Figure 6:**
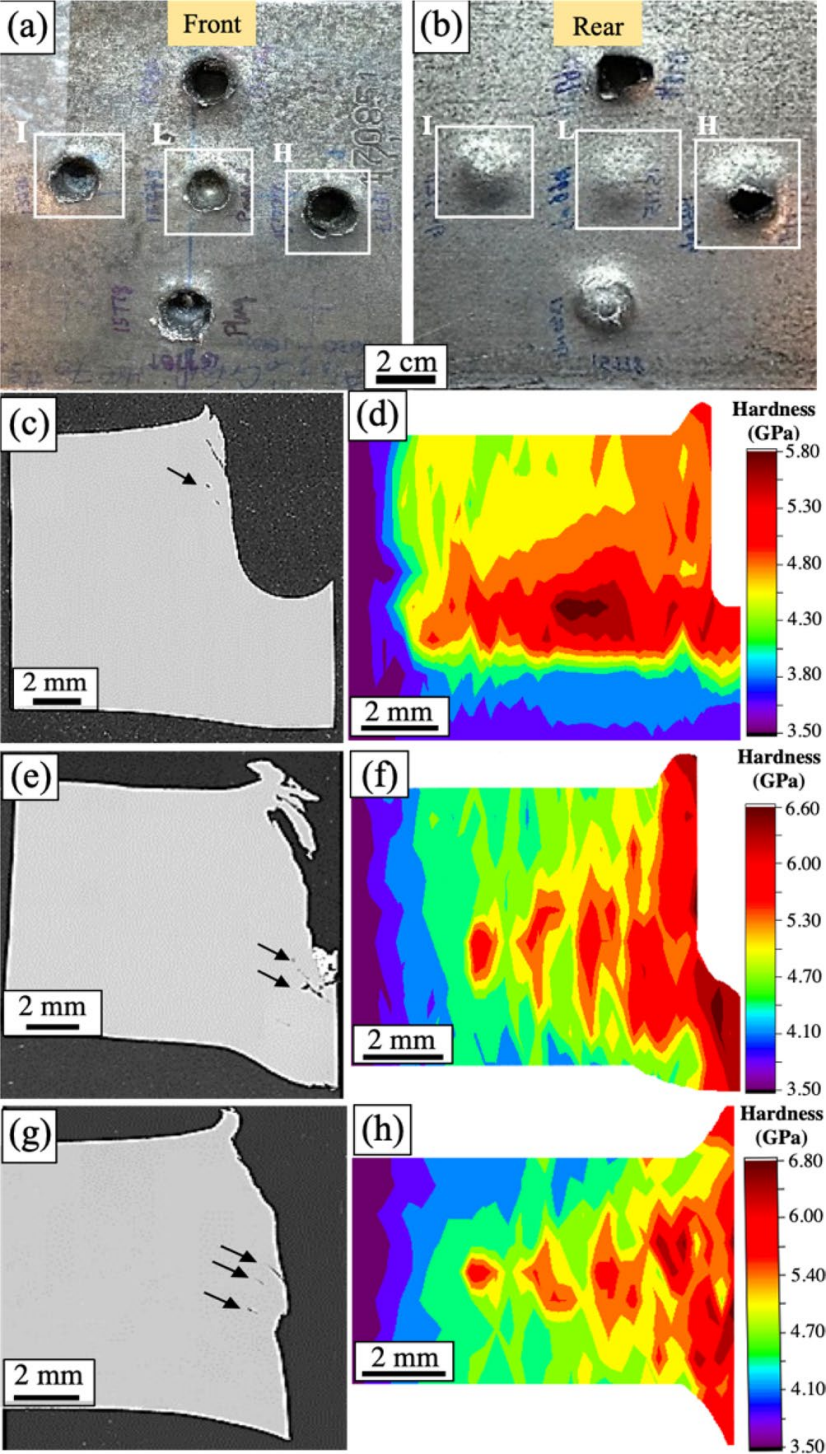
Ballistic impact and hardness contour maps: (**a**) front face and (**b**) rear face of the target plate showing the three shot conditions. Low velocity impact of 1066 m/s: (**c**) cross-sectional SEM image and (**d**) corresponding hardness contour map; Intermediate velocity impact of 1340 m/s: (**e**) cross-sectional SEM image and (**f**) corresponding hardness contour map; High velocity impact of 1465 m/s: (**g**) cross-sectional SEM image showing ductile hole growth failure with parallelly spaced macro-cracks indicated by black arrows and (**h**) corresponding hardness contour map.

The front and rear faces of the target plate after ballistic impact are shown in Fig. 6a and b, respectively. Three shot conditions were identified depending on the impact velocity: low velocity impact (L), intermediate velocity impact (I), and high velocity impact (H). Low and intermediate velocity impact exhibited partial penetration whereas high velocity impact exhibited complete penetration. The front face showed typical crater lip formation for all three impact conditions due to unconfined plastic flow of material towards the surface as the projectile penetrated through the target plate. The size of the lip increased with increasing impact velocity and was maximum for the high velocity impact condition. A smooth bulge without any macroscopic cracks was seen on the rear face of the target for low and intermediate velocity impact conditions as shown in Fig. 6b. High velocity impact showed a smooth exit hole with large petal formation. This indicates ductile hole growth type of failure mode, which is typically seen in target materials with good ductility^11,37^. During ductile hole growth, the target material absorbs the kinetic energy from the projectile through plastic deformation and maximizes the resistance to projectile penetration for a given strength level^11,37^. A cross-section SEM image of the low velocity impact in Fig. 6c shows crater formation with macro-cracks emanating along the crater wall as indicated by an arrow. Figure 6d shows the nanoindentation hardness contour map for low velocity condition. The hardness values were highest in the region close to the crater wall with maximum hardness in the deformed region of ~ 5.8 GPa compared to the undeformed region hardness of 3.5 GPa, which indicates substantial work-hardening of the alloy. The affected area after the projectile impact was found to be ~ 8.5 mm in the horizontal direction and about 3 mm in the vertical direction below the crater bottom. High values of hardness were observed in the region near the lip (entry point), which is likely because this region underwent intense deformation resulting in a microstructure that consisted of highly deformed grains and microbands. At the intermediate impact velocity, macro-cracks were seen near the region where the projectile was arrested, as shown by black arrows in Fig. 6e. Cracking may have occurred after the projectile was arrested, as regions of intense shearing beneath the projectile would be rapidly quenched once the projectile stopped, resulting in a high-strength and brittle microstructure. The nanoindentation hardness contour map for the intermediate velocity condition is shown in Fig. 6f. The maximum hardness in the deformed region (~ 6.6 GPa) was almost twice the undeformed region hardness (~ 3.5 GPa). The hardness values were highest in the region parallel to the crater wall and beneath the projectile. The affected region was ~ 6.5 mm in the radial direction from the crater wall in this condition compared to ~ 8.5 mm for low velocity impact condition. Similar to the low velocity impact, the hardness decreased with increasing distance from the crater wall. The high velocity impact showed ductile hole enlargement failure with parallelly spaced macro-cracks originating from the crater wall as shown by arrows in Fig. 6g. A nanoindentation hardness contour map for high velocity impact is shown in Fig. 6h. The maximum hardness in the deformed region was ~ 6.8 GPa compared to the undeformed region hardness of ~ 3.5 GPa, and the deformed region was ~ 6 mm in radial direction from the crater wall. The hardness values were high in the regions parallel to the crater wall and highest near the entry and exit regions due to intense, unconfined plastic flow in these regions. This condition showed the highest increase in the hardness among the three impact conditions and the values progressively decreased with increase in distance from the crater wall.

### Microstructural characterization after ballistic impact

**Figure 7:**
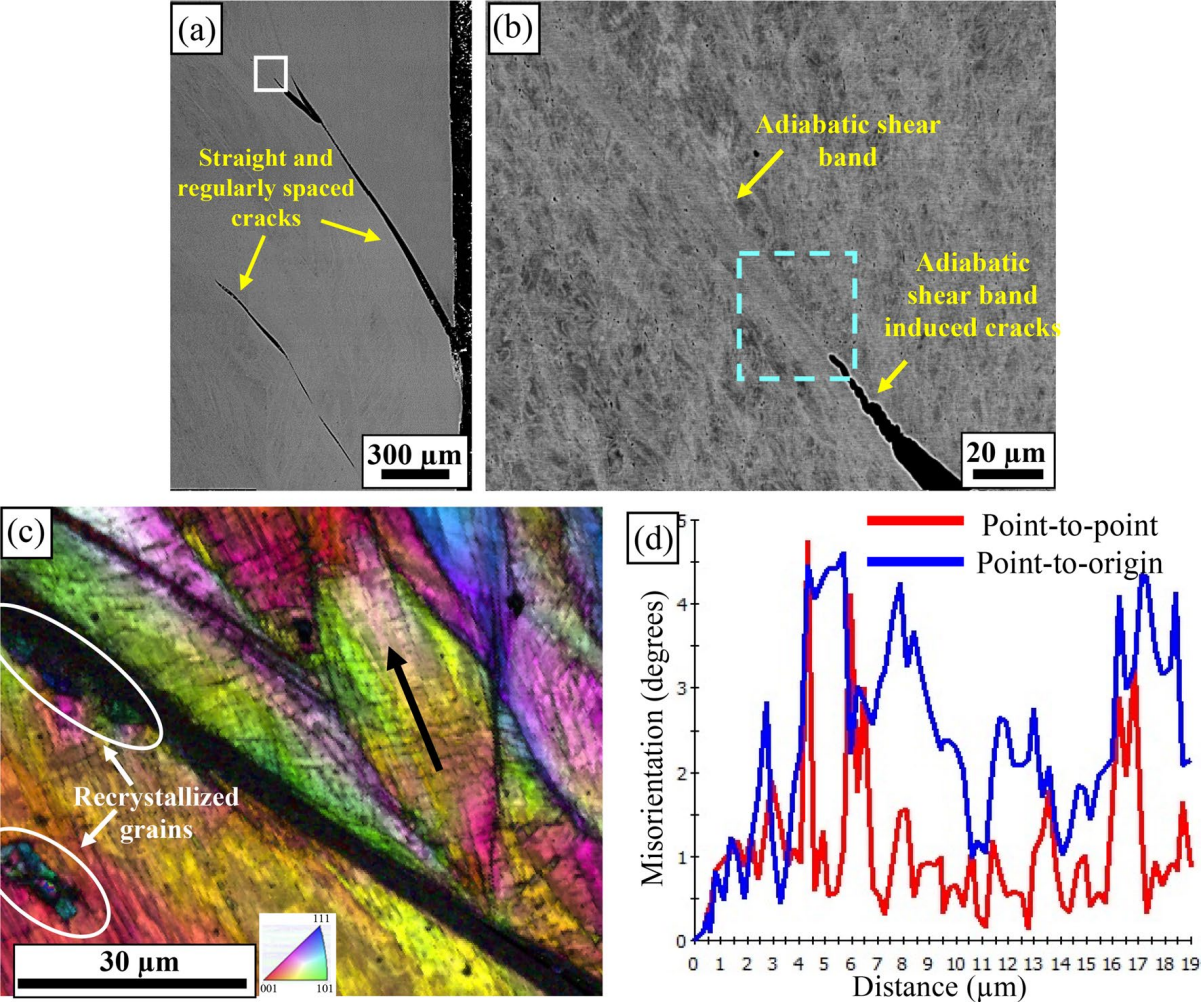
Microstructural characterization after high velocity ballistic impact of 1465 m/s: (**a**) low magnification SEM image showing parallelly propagating cracks and (**b**) backscattered SEM image of region shown with a white box in (**a**) shows ASBs and ASB induced crack; (**c**) IPF + IQ image of region shown with dotted box indicates recrystallized grains near the ASB and high density of microbands in the deformed grains; (**d**) misorientation profile of microbands along the black arrow shown in (**c**) indicates an average misorientation of ~ 3–4 degrees.

A low magnification SEM image of high velocity impact condition is shown in Fig. 7a. Regularly spaced macro-cracks are seen emanating from the crater wall and propagating inside the target material along the radial direction. These cracks did not show any bifurcations along their path and helped in the smooth penetration of projectile through the target plate which corresponds to the large section of deformed region demonstrated by the nanoindentation hardness map. Any bifurcations would have led to the formation of interconnected network of cracks and may have resulted in premature failure of the target. A high-magnification backscattered SEM image is shown in Fig. 7b (white box of Fig. 7a) revealing ASBs and ASB-induced cracking. The ASB was observed to be ~ 3–4 µm wide. During ballistic impact, the target material experiences a complex deformation history and, in some cases, may undergo severe local strains up to ~ 500%^38^. In contrast to uniform deformation during SHPB experiments, strain gradients are naturally imposed on the target by the projectile during ballistic impact and promote the formation of localized bands of deformation. For similar strains and strain rates, deformation banding is likely to be more prominent during ballistic impact due to the inhomogeneous nature of deformation compared with SHPB experiments^39,40^. An IPF + IQ image of an area close to the ASB (Fig. 7c) showed narrow regions of recrystallized grains of different orientations with average grain size of ~ 1 µm, possibly formed due to significant dynamic recrystallization after quenching near the ASB from the temperature rise during plastic deformation^11^. Formation of ultrafine grains near/inside the ASBs because of dynamic recrystallization has been previously reported^14,40,41^. As the deformation proceeds during ballistic impact, the density of dislocations significantly increases inside the dynamically recrystallized grains. This promotes the formation of voids and their coalescence, ultimately resulting in crack nucleation and growth^42^. The cracks nucleate inside the ASBs with tensile stresses acting perpendicular to the shear surfaces as the projectile penetrates through the target and assists in premature failure of the target at high strain rates^14,43^. Highly deformed grains near the ASBs showed high density of microbands. The misorientation of these microbands along the black arrow in Fig. 7c was found to be ~ 3–4 degrees, as seen in Fig. 7d, indicating that there was no evidence of twinning at this length-scale.

Geometry of the propagating shock wave defines the deformation mode in alloys under ballistic impact. For example, in impact cratering involving spherical shock waves similar to the current study, metals with low SFE showed micro-twins or a combination of microbands and micro-twins^44^. In FCC alloys with low SFE, the presence of planar dislocation arrays promotes the formation of microbands^42^, while twins may form due to dissociated dislocations and decrease the driving force for microband formation^44^. Hence, there is a competition between the formation of microbands and twins in low SFE materials at high strain rates. However, there was no evidence of twin formation in the current study after ballistic impact possibly due to the complex SFE landscape of our alloy from the heterogeneous microstructure.

**Figure 8:**
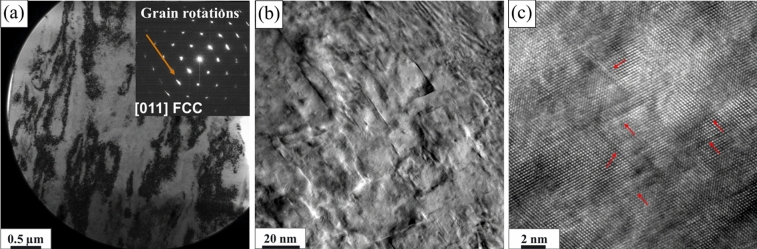
TEM characterization of the sample from an area close to the crater wall after high velocity ballistic impact of 1465 m/s: (**a**) Low magnification BF-TEM image showing high density of dislocation bands with the inset showing SADP pattern taken along [011]_FCC_ ZA suggesting grain boundary rotation as marked with a red arrow and absence of L1_2_ super lattice reflections; (**b**) BF-TEM and (**c**) HRTEM images showing high density of stacking faults.

To get more insights into the deformation mechanism for the ballistic strain rates, TEM characterization was done close to the crater wall. The BF-TEM image shown in Fig. 8a indicates dense dislocation bands throughout due to intense deformation during ballistic impact. The inset in Fig. 8a shows SADP from [011]_FCC_ ZA suggesting grain rotation as marked with an arrow. It also shows the loss of superlattice spots previously indicated on Figs. 2b and 5b suggesting loss of L1_2_ ordering in the matrix. Figure 8b and c show the BF-TEM and HRTEM image of the impacted region indicating very high density of stacking faults. Therefore, the overall deformation mechanism during ballistic impact of our alloy was characterized by the formation of high density of stacking faults, grain rotation, and disordering of L1_2_ phase.

## Summary

In summary, the microstructural features and mechanical response of Al_0.3_CoCrFeNi high entropy alloy was characterized in a wide range of strain rates including quasi-static deformation, Split-Hopkinson pressure bar testing, and ballistic impact. The alloy exhibited an excellent combination of strength and ductility from the bimodal microstructure, nano-scale L1_2_ phase in FCC matrix and grain-boundary B2 precipitates. The alloy exhibited a high strain rate sensitivity. The microstructure after quasi-static and SHPB strain rates showed highly deformed grains with deformation bands and small density of stacking faults at dynamic strain rate, without any evidence of shear localization and twins. The suppression in shear localization was attributed to the excellent strain hardenability of the alloy. The target material failed by ductile hole growth in ballistic impact and the deformed microstructure was characterized by ASBs, ASB induced cracks, microbands, and dynamically recrystallized ultrafine grains near ASBs. Nanoindentation hardness maps showed hardness increase by a factor of two in the deformed region compared to undeformed region. The hardness values were high in the regions parallel to the crater wall and highest near the entry and exit regions due to intense plastic deformation. Microstructure of the alloy after ballistic impact showed high density of dislocations, profuse stacking faults, grain boundary rotation, and disordering of L1_2_ phase.
